# Metabolic and inflammatory parameters in relation to baseline characterization and treatment outcome in patients with prolactinoma: insights from a retrospective cohort study at a single tertiary center

**DOI:** 10.3389/fendo.2024.1363939

**Published:** 2024-04-05

**Authors:** Susanna Hofbauer, Laura Horka, Samuel Seidenberg, Raffaele Da Mutten, Luca Regli, Carlo Serra, Felix Beuschlein, Zoran Erlic

**Affiliations:** ^1^ Department of Endocrinology, Diabetology and Clinical Nutrition, University Hospital Zurich (USZ) and University of Zurich (UZH), Zurich, Switzerland; ^2^ Department of Neurosurgery, Clinical Neuroscience Center, University Hospital Zurich (USZ) and University of Zurich (UZH), Zurich, Switzerland

**Keywords:** prolactinoma, pituitary, hypopituitarism, metabolism, inflammation, treatment, prognosis

## Abstract

**Background:**

Prolactinomas (PRLs) are prevalent pituitary adenomas associated with metabolic changes and increased cardiovascular morbidity. This study examined clinical, endocrine, metabolic, and inflammatory profiles in PRL patients, aiming to identify potential prognostic markers.

**Methods:**

The study comprised data from 59 PRL patients gathered in a registry at the University Hospital of Zurich. Diagnostic criteria included MRI findings and elevated serum prolactin levels. We assessed baseline and follow-up clinical demographics, metabolic markers, serum inflammation-based scores, and endocrine parameters. Treatment outcomes were evaluated based on prolactin normalization, tumor shrinkage, and cabergoline dosage.

**Results:**

The PRL cohort exhibited a higher prevalence of overweight/obesity, prediabetes/diabetes mellitus, and dyslipidemia compared to the general population. Significant correlations were found between PRL characteristics and BMI, HbA1c, and fT4 levels. Follow-up data indicated decreases in tumor size, tumor volume, prolactin levels, and LDL-cholesterol, alongside increases in fT4 and sex hormones levels. No significant associations were observed between baseline parameters and tumor shrinkage at follow-up. A positive association was noted between PRL size/volume and the time to achieve prolactin normalization, and a negative association with baseline fT4 levels.

**Conclusion:**

This study underscores the metabolic significance of PRL, with notable correlations between PRL parameters and metabolic indices. However, inflammatory markers were not significantly correlated with patient stratification or outcome prediction. These findings highlight the necessity for standardized follow-up protocols and further research into the metabolic pathogenesis in PRL patients.

## 1 Introduction

Pituitary adenomas are frequent intracranial tumors following only meningiomas and gliomas in their incidence (1). Prolactinomas (PRLs) are the most common clinical subtype among pituitary adenomas (2). Their prevalence and incidence is about 50 per 10.000 and 3-5 new cases per 100.000 population per year, according to newer epidemiological studies (3).

The clinical signs and symptoms of PRL are mainly related to hyperprolactinemia, which is the hallmark of these tumors (4). In general, prolactin levels correlate well with pituitary adenoma size (5–7). Rarely, these tumors are symptomatic due to the mass effect causing compression of the nearby structures, resulting in primarily visual loss or pituitary insufficiency. The most common and known clinical presentation of hyperprolactinemia is hypogonadism, related to the inhibition of the gonadotropin secretion and action (8). However, there is increasing evidence of metabolic alterations in patients with hyperprolactinemia, which might be related to the increased cardiovascular morbidity observed in patients with high prolactin levels (9). Changes in lipid and glucose metabolism and weight gain has been described (10–18). Whilst some of the effects are related to the concomitant hypogonadism (19), others might be directly evoked by the prolactin hypersecretion itself or other unknown mechanism [reviewed by (20, 21)].

Medical treatment with dopamine agonists (DA) is the therapy of choice for PRL with humoral response, defined as normoprolactinemia in 68% of cases, tumor shrinkage in 62% of cases and relieving infertility or other symptoms in 53%, respectively (22, 23). Recurrence of hyperprolactinemia after withdrawal of DAs varies widely among different studies between 2- 80%, depending of the DA-type, treatment duration and initial tumor size (24, 25). For patients who are intolerant or resistant to DA, surgery is the best option. With recent advances in neurosurgical strategies, treatment related morbidity and mortality has decreased significantly, and it is considered by some specialists to be a valid first-line therapeutic alternative (26, 27), since the surgical cure is seen in up to 67% of patients (27).

As response to medical treatment varies considerably between patients, identifying new markers for diagnostic stratification and prognosis would aid in identifying patients in need of more aggressive medical treatments or even surgery as a first option. Both metabolic as well as inflammatory markers have been applied successfully as diagnostic and prognostic markers in tumor patients, including patients with endocrine tumors (28–32). In these conditions (e.g. primary aldosteronism, catecholamine excess) metabolic comorbidities have been described as well (33, 34) and metabolic markers have shown potential for diagnostic purposes (30–32).

Several inflammatory markers have been studied in tumor patients. The Neutrophile-to-Lymphocyte-Ratio (NLR) as an inflammatory marker reflects an ineffective immune response to the tumor and invasiveness with poor outcomes. The Platelet-to-Lymphozyte-Ratio (PLR) is also associated with poor cancer outcomes. The Glasgow Prognostic Score (GPS) is reflecting malnutrition and systemic inflammation. The Systemic Immune Inflammation Index (SII) is an important prognostic factor associated with lower postoperative survival in several types of cancer (35). A poor cancer prognosis is often associated with a reduced Prognostic Nutrition Index (PNI) (36). The Neutrophil-Platelet Score (NPS) have a prognostic value in different tumor diseases (37). Whilst metabolic changes in patients with Cushing syndrome and acromegaly are part of the syndrome description, increased inflammation is not a well-acknowledged component (38, 39). For decades, we have known that there is an increased inflammation in patients with Cushing syndrome and that this might contribute to cardiovascular morbidities in patients with Cushing syndrome (40). Similarly, in patients with acromegaly, proinflammatory processes have been described which influence the cardiovascular risk profile of these patients before and after treatment (41). Therefore is of no surprise, that recent studies evidenced increased inflammatory markers in patients with pituitary adenomas, in specific patients with Cushing disease, and with much less extent in acromegaly and PRL patients compared to non-functioning adenoma (28, 29) To our knowledge, only one study focused on this topic in patients with PRL, who found some differences in the hemostatic parameters in comparison to healthy controls (42).

Consequently, our study aimed to investigate the potential of metabolic and inflammatory changes in patients with PRL, both for characterizing their condition and as a prognostic tool.

## 2 Materials and methods

### 2.1 Patients

PRL patients from the Network of Excellence for Neuroendocrine Tumours (NeoExNET) Registry of the University Hospital of Zurich (USZ) were included in this study. The NeoExNET Registry encompassed all patients aged over 18 years, diagnosed with pituitary adenoma, who provided informed consent for the use of their retrospective and prospective clinical, radiological, laboratory, and, when available, histological data.

For this study, the diagnosis of PRL was based by fulfilling two criteria: First, pituitary lesion meeting the criteria for adenoma in magnetic resonance imaging and second, serum prolactin levels exceeding 30 µg/l after excluding macroprolactinemia, where clear distinction between PRL and hormonally inactive adenoma with stalk effect hyperprolactinemia was possible. This was determined after evaluating adenoma size and prolactin level, as well as the morphological response in MRI following treatment, if available.

Patients were excluded if the diagnosis could not be confirmed according to the above criteria, if there were missing baseline data before the start of DA treatment, or if they had rheumatological diseases, infections, or concurrent other tumor diseases.

### 2.2 Clinical and laboratory parameters

We examined the endocrine and metabolic patterns, as well as inflammation-based scores, to characterize baseline attributes and assess treatment outcomes in PRL patients. The evaluated parameters included baseline measurements (before cabergoline treatment) and follow-up data (after achieving prolactin normalization with treatment). For follow-up, we considered the earliest data available post-prolactin normalization or, in cases where normoprolactinemia was not attained, data after one year of treatment (with one exception included after 2.5 years). We incorporated clinical and demographic data (BMI, blood pressure, heart rate, age, sex), metabolic markers (total cholesterol, LDL-cholesterol, HDL-cholesterol, triglycerides, HbA1c), serum inflammation-based scores (35) [NLR, PLR, GPS, NPS (37), SII (43), PNI (36)], endocrine plasma/serum parameters (fT4, sex hormones [testosterone for men, estradiol for women], cortisol, prolactin, IGF1), and imaging data [PRL size as maximum diameter and volume, and PRL volume according to a previously described formula (44)]. Details on the formula used for calculation of the inflammatory scores and volume are listed in the **Supplementary Material**.

For assessing treatment outcomes, we focused on the following parameters: a) achievement of normoprolactinemia (yes/no), b) time taken to achieve prolactin normalization (in days), c) dosage of cabergoline required to reach normoprolactinemia (in mg, and d) percentage of tumor shrinkage.

The time to prolactin normalization was determined as the first instance of documented prolactin normalization. This time might differ from the follow-up measurement, which was taken as the first complete data set available from the point of prolactin normalization. Due to the limited sample size, we were unable to include in our study the analysis of remission post-treatment withdrawal (only 9 patients from the registry completed treatment) or resistance to pharmacological treatment (4 patients did not achieve normoprolactinemia after one year, and 1 patient after 2.5 years of dopamine agonist treatment).

### 2.3 Statistical analysis

#### 2.3.1 Description of the cohort at baseline and follow up

Patient characteristics and comorbidities were summarized as follows: frequencies for categorical variables, means with 95% confidence intervals for normally distributed variables, and medians with minimum and maximum values for non-normally distributed variables, as determined by the Shapiro-Wilk test. To identify differences between groups (microprolactinoma and macroprolactinoma) at baseline and follow-up, we used the Pearson Chi-squared test or the Fisher exact test for sample sizes less than 50 for categorical variables. For numerical variables, the t-test was applied to normally distributed data, and the Mann-Whitney U test for non-normally distributed data. A p-value of ≤0.05 was considered statistically significant.

#### 2.3.2 Association analysis between prolactinoma parameters and the measured metabolic and inflammatory parameters at baseline

We employed the Spearman test to assess correlations between what we defined as PRL parameters - specifically PRL size, PRL volume, and prolactin level - and the clinical, metabolic, endocrine data, as well as serum inflammation-based scores measured at baseline. A p-value of ≤0.05 was set as the threshold for significance. Variables showing significant correlations were further examined using univariate logistic regression analysis. Due to a notable correlation between prolactin and sex, and adenoma size with age (as shown in **Supplementary Table 1**), we conducted a secondary analysis that included these variables in the regression model for those clinical, metabolic, inflammatory, and endocrine variables significantly correlated with sex and/or age (referenced in **Supplementary Table 2**). For the adjusted regression analysis results concerning age/sex, we considered a significance p-value of ≤0.05 after applying the Bonferroni correction.

#### 2.3.3 Association analysis between prolactinoma and the measured parameters at follow up

In the subgroup of patients with follow-up data, we evaluated the differences in clinical, metabolic, endocrine, and inflammatory parameters from baseline to follow-up. For this analysis, the paired-samples t-test was used for normally distributed parameters, and the Wilcoxon signed-rank test for non-normally distributed parameters, with a significance threshold set at a p-value of ≤0.05. Additionally, we performed an exploratory correlation analysis to investigate the relationship between the changes (delta) in PRL parameters and the deltas in metabolic and inflammatory parameters. For those parameters showing significant correlations, logistic regression analysis was conducted to assess their association. Furthermore, we analyzed the correlation between baseline PRL parameters and the changes (delta) in endocrine, metabolic, and inflammatory parameters, and those with significant correlations were subsequently examined for association in the regression analysis.

#### 2.3.4 Outcome prediction

We conducted a univariate regression analysis to explore the relationship between treatment outcome markers (time to prolactin normalization, tumor shrinkage) as dependent variables and the clinical, metabolic, and serum inflammation-based scores at baseline as independent variables. For tumor shrinkage, the results were further adjusted for the time interval between the baseline and follow-up MRI scans. However, due to a lack of independence in residuals, as indicated by Durbin-Watson statistics being less than 1.0, we were unable to perform regression analysis for the cabergoline dosage required to achieve normoprolactinemia.

All statistical analyses were carried out using SPSS software, version 26 (IBM).

## 3 Results

Out of the 90 PRL patients in the NeoExNET registry, 31 were excluded from the study. Twenty-five were excluded due to incomplete baseline laboratory levels, one for undergoing surgical treatment, and five because they did not meet the distinct criteria for differentiating between non-functioning pituitary adenoma and PRL. Additionally, patients with primary hypothyroidism, whether or not they were undergoing levothyroxine substitution (a total of 4 patients), and those on oral contraceptive pills or testosterone substitution (a total of 2 patients) at the time of PRL diagnosis were also excluded. An exception was made for one patient who had received a single dose of testosterone enanthate two weeks before the baseline laboratory tests; this patient was included in the study.

Baseline characteristics of the remaining 59 patients are detailed in **Table 1A**. This group included 12 patients with microprolactinoma (33% women, average age 36 years) and 47 patients with macroprolactinoma (49% women, average age 34 years). There were no significant differences in age and sex between microprolactinoma and macroprolactinoma patients. Hyperprolactinemia was present in all patients, with 47 patients (89%) exhibiting hypogonadism at presentation, more commonly in those with macroprolactinoma. Other forms of pituitary insufficiency were found in 12 (27%) of the macroprolactinoma patients (corticotrop 7%, thyreotrop 20%) but not in any microprolactinoma patients. Symptoms of mass effect, particularly visual disturbances, were noted in six macroprolactinoma patients but in none with microprolactinoma.

**Table 1A:** Baseline characteristics of patients with micro- and macroprolactinoma.

	Baseline		
	Microprolactinoma	Macroprolactinoma	p-value
Total number of patients	12	47	
Sex (f/m)	4/8	23/24	0.333
Age (years)*	36.5 (20.0-64.0)	34.0 (16.0-70.0)	0.814
Adenoma size (mm)*	7.0 (5.0-9.9)	18.4 (10.0-61.0)	<0.001
Adenoma volume (ml)*	0.14 (0.03-0.51)	1.53 (0.28-80.71)	<0.001
BP systolic (mmHg) *Missing data (n)*	125 (115-136) *1*	124 (119-129) *4*	0.817
BP diastolic (mmHg)* *Missing data (n)*	78 (68-95) *1*	81 (60-110) *4*	0.675
Heart rate (bpm)* *Missing data (n)*	68.5 (50.0-94.0) *0*	72.0 (54.0-108.0) *7*	0.361
BMI (kg/m^2^) *Missing data (n)*	26.2 (22.8-29.7) *0*	28.1 (25.9-30.3) *1*	0.415
HbA1c (%)* *Missing data (n)*	5.5 (4.9-6.) *5*	5.5 (4.7-9.9) *23*	0.627
Total Cholesterol (mmol/l) *Missing data (n)*	4.7 (3.6-5.8) *5*	4.9 (4.3-5.5) *32*	0.762
LDL (mmol/l) *Missing data (n)*	2.9 (2.0-3.8) *5*	3.1 (2.5-3.6) *33*	0.726
HDL (mmol/l) *Missing data (n)*	1.3 (0.9-1.7) *5*	1.2 (1.1-1.3) *34*	0.503
TG (mmol/l)* *Missing data (n)*	0.9 (0.4-2.2) *5*	1.3 (0.5-3.6) *32*	0.298
Prolactin (ug/l)* *Missing data (n)*	92.0 (30.3-254.7) *0*	331.0 (30.2-4700.0) *0*	0.001
fT4 (pmol/l)* *Missing data (n)*	14.6 (12.4-17.7) *0*	13.9 (4.3-18.7) *0*	0.178
Cortisol (nmol/l)* *Missing data (n)*	383.0 (153-483) *1*	291.0 (25-736) *3*	0.115
Estradiol (pmol/l)* *Missing data (n)*	102.0 (1.9-110.0) *9*	80.0 (0.0-623.0) *26*	1.000
Testosterone (nmol/l)* *Missing data (n)*	9.5 (6.8-22.2) *5*	5.6 (0.0-23.0) *24*	0.005
IGF1 (ug/l) *Missing data (n)*	173.0 (144.7-201.3) *0*	179.9 (154.6-205.1) *7*	0.775
NLR* *Missing data (n)*	1.7 (1.0-2.4) *2*	1.7 (0.7-9.9) *15*	0.919
PLR* *Missing data (n)*	138.9 (88.5-182.6) *2*	115.6 (60.7-413.3) *15*	0.390
PNI *Missing data (n)*	55.7 (53.6-57.9) *3*	55.4 (52.8-58.1) *21*	0.895
SII* *Missing data (n)*	386.3 (283.2-783.5) *2*	418.4 (183.7-2463.5) *15*	0.965
NPS (0/1/2) *Missing data (n)*	9/0/0 *3*	31/1/0 *15*	0.591
GPS (0/1/2) *Missing data (n)*	4/0/0 *8*	12/1/1 *33*	0.725
Dyslipidemia (yes) *Missing data (n)*	3 *0*	9 *0*	0.653
Statine treatment (yes)	2	1	
Hypertension (yes) *Missing data (n)*	1 *0*	6 *0*	0.672
AH treatment (yes) *Missing data (n)*	0 0	4 0	0.295
Obesity (yes) *Missing data (n)*	3 *0*	15 *1*	0.612
Prediabetes (yes) *Missing data (n)*	1 *0*	6 *0*	0.672
Diabetes *Missing data (n)*	1 *0*	4 *0*	0.984
Corticotropic deficiency (yes)** *Missing data (n)*	0 *0*	3 *0*	0.369
Thyrotropic deficiency (yes)** *Missing data (n)*	0 *0*	9 *0*	0.100
Somatotropic deficiency (yes)** *Missing data (n)*	0 *0*	0 *0*	
Hypogonadism (yes)** *Missing data (n)*	7 *0*	40 *0*	0.040

Values are indicated as median with range in brackets for not normally distributed numerical variables, mean with 95% confidence interval in brackets for normally distributed numerical variables, frequencies for categorical variables.

For sex hormones, testosterone was only evaluated in men and estradiol only in women.

n, indicates the number of patients (frequency), BP, Blood pressure.

*not normally distributed numerical variables.

**all patients with corticotropic and thyrotrophic insufficiency were under hormonal replacement treatment with hydrocortisone and levothyroxine respectively; with the exception of one male patient who received a single dose of 250mg of testosterone enantate 2 weeks prior baseline measurements, all patients with hypogonadism were not under hormonal replacement treatment.

At baseline, macroprolactinoma patients had lower testosterone levels (p=0.005) and higher prolactin levels (p<0.001) compared to those with microprolactinoma. No other differences in clinical, endocrine, metabolic, and inflammatory parameters were observed. A negative correlation was identified between adenoma size, volume, and prolactin level with testosterone levels (**Supplementary Table 3**). The absence of correlation with estradiol levels might be due to non-standardized sample collection relative to the menstrual cycle. However, a significant negative correlation with fT4 was noted for all three PRL parameters. This association was confirmed after adjusting for age and sex in the regression analysis. Additionally, a positive correlation was found between adenoma size and BMI and HbA1c, adenoma volume and BMI, as well as prolactin with heart rate, BMI, and HbA1c (**Supplementary Table 3**). Of these correlations, only the association between prolactin and HbA1c was confirmed in the regression analysis (**Table 1B**).

**Table 1B:** Regression analysis results between PRL parameters and the significant metabolic features from the correlation analysis.

	Adenoma size		*adjusted by age*		Adenoma Volume		Prolactin		*adjusted by age*	
	*beta*	*p-value*	*beta*	*p-value*	*beta*	*p-value*	*beta*	*p-value*	*beta*	*p-value*
**BMI**	0.244	0.065	0.205	0.136	0.156	0.242	0.236	0.075	0.154	0.289
**Heart rate**							0.043	0.762		
**HbA1c**	0.269	0.143	0.155	0.342			0.511	**0.003**		

Univariate regression analysis, as detailed in the methods section, was conducted. Additionally, a multivariate regression analysis that included age was performed for assessing the relationship of BMI with adenoma size and prolactin, and of HbA1c with adenoma size. ‘Beta’ denotes the standardized regression coefficient. Results highlighted in bold indicate statistical significance, corresponding to a p-value of ≤0.05.

### 3.1 Clinical, endocrine, metabolic and inflammatory changes under treatment

Follow-up data were available for 49 patients. However, for further statistical analyses, we excluded three patients: two who underwent surgical treatment after the baseline visit and one who was pregnant at follow-up. Consequently, 46 patients were included in the follow-up analyses (**Table 2**). The median duration between baseline and follow-up data collection was 579 days (ranging from a minimum of 44 days to a maximum of 4292 days). The initial differences in prolactin and testosterone levels observed between micro- and macroprolactinoma patients were no longer present at follow-up, as anticipated. However, differences in tumor size and volume between the two groups persisted at follow-up.

**Table 2:** Characteristics of patients with micro- and macroprolactinoma at follow up as well as differences from baseline at time of prolactin normalization.

	Follow Up			Differences from Baseline	
	Microprolactinoma	Macroprolactinoma	p-value	delta	p-value
Total number of patients	7	39			
Sex (f/m)	2/5	20/19	0.418		
Adenoma size (mm)* *Missing data (n)*	5.3 (0.0 – 9.0) *1*	15.1 (0.0-30.6) *2*	0.028	-4.0 (-36.0;10.6) §	<0.001
Adenoma volume (ml)* *Missing data (n)*	0.03 (0.0-0.23) *1*	0.59 (0.0-8.57) *2*	0.026	-0.68 (-79.60; 0.19)§	<0.001
BP systolic (mmHg) *Missing data (n)*	107 (83-146) *0*	123 (107-167) *1*	0.157	-1.4 (-6.3; 3.5)	0.576
BP diastolic (mmHg) *Missing data (n)*	73.6 (63.0-84.1) *0*	82.9 (78.6-87.2) *1*	0.088	0.6 (-3.0; 4.2)	0.742
Heart rate (bpm) *Missing data (n)*	74.1 (58.4-89.9) *0*	76.4 (72.6-80.2) *1*	0.749	2.4 (-2.0; 6.7)	0.274
BMI (kg/m^2^)* *Missing data (n)*	26.9 (21.0-37.0) *1*	28.0 (18.0-51.0) *6*	0.835	0.3 (-1.1; 1.8)	0.632
HbA1c (%) *Missing data (n)*	5.4 (4.8-5.9) *0*	5.4 (5.2-5.5) *13*	0.980	-0.1 (-3.9; 0.6)§	0.060
Total Cholesterol* (mmol/l) *Missing data (n)*	4.5 (3.6-5.5) *0*	4.7 (4.1-5.2) *19*	0.808	0.7 (-1.7; 0.3)	0.172
LDL (mmol/l) *Missing data (n)*	2.7 (1.8-3.5) *0*	2.7 (2.3-3.1) *19*	0.999	-0.4 (-3.7; 0.3)§	0.020
HDL (mmol/l)* *Missing data (n)*	1.2 (1.0-1.7) *0*	1.3 (0.9-2.7) *19*	1.000	-0.04 (-0.15; 0.07)	0.428
TG (mmol/l)* *Missing data (n)*	1.1 (0.6-4.2) *0*	1.1 (0.4-2.1) *19*	0.850	-0.2 (-0.8; 0.4)	0.469
Prolactin (ug/l)* *Missing data (n)*	6.7 (0.9-50.6) *0*	9.1 (0.0-241.0) *0*	0.632	-246.6 (-4699.1; 28.6)§	<0.001
fT4 (pmol/l)* *Missing data (n)*	15.6 (12.7-17.9) *0*	14.8 (12.1-24.8) *1*	0.529	1.1 (-2.6; 10.7)§	0.004
Cortisol (nmol/l)* *Missing data (n)*	310.5 (212.0-490.0) *1*	274.0 (188.0-609.0) *2*	0.504	7.0 (-489.0; 510.0)§	0.928
Estradiol (pmol/l)* *Missing data (n)*	203.0 (124.0-419.0) *4*	291.0 (0.0-2379.0) *20*	0.651	218.5 (-351.0; 2295.0)§	0.011
Testosterone (nmol/l) *Missing data (n)*	14.9 (10.5-19.2) *3*	13.8 (9.4-18.1) *24*	0.790	7.0 (3.8; 10.2)	<0.001
IGF1 (ug/l) *Missing data (n)*	250.2 (135.9-364.4) *3*	187.2 (146.2-228.2) *21*	0.175	27.5 (-3.2; 58.2)	0.076
NLR* *Missing data (n)*	1.6 (1.1-2.9) *2*	1.7 (0.6-9.3) *4*	0.781	-0.05 (-1.95; 4.18)§	0.964
PLR* *Missing data (n)*	113.0 (79.7-166.0) *2*	118.7 (49.1-490.9) *4*	0.721	6.8 (-12.5; 26.1)	0.475
PNI *Missing data (n)*	52.7 (0-60.3) *2*	55.1 (10.7-70.7) *5*	0.921	-1.1 (-2.5; 0.3)	0.105
SII* *Missing data (n)*	326.3 (293.6-740.4) *2*	405.5 (133.3-2157.0) *4*	0.449	-36.9 (-450.7; 1121.5)§	0.616
NPS (0/1) *Missing data (n)*	5/0 *2*	30/2 *7*	1.000		
GPS (0/1/2) *Missing data (n)*	4/0/0 *3*	16/3/0 *20*	0.250		
Time between baseline and follow up data measurement	308 (133 – 1073)	602 (44-4292)			
Tumorshrinkage (%)* *Missing data (n)*	58.3 (0.0-100.0) 1	69.3 (-20.4-100) 2	0.771		
Time between baseline and follow up MRI (days)*	753.5 (173-1199)	702.0 (197-4442)	1.000		
Normalization of prolactin (days)* *Missing data (n)*	121 (30-456) 1	121 (15-1460) 5	0.726		
Cabergoline dosage (mg/week) at time of prolactin normalization*	0.5 (0.5-1.0)	0.5 (0.25-7)	0.894		

Values are indicated as median with range in brackets for not normally distributed numerical variables, mean with 95% confidence interval in brackets for normally distributed numerical variables, frequencies for categorical variables.

For sex hormones, testosterone was only evaluated in men and estradiol only in women.

n, indicates the number of patients (frequency), BP, Blood pressure.

*not normally distributed variables.

§ not normally distributed difference between baseline and follow up.

All patients with corticotropic (three) and thyrotropic (nine) deficiencies were under replacement therapy at baseline. Of the three patients, only one had persistent corticotropic insufficiency at follow-up under the same dosage of hydrocortisone replacement (20mg), but all patients with thyrotropic deficiency were still under substitution treatment at follow-up evaluation without a significant change (p=0.705 according to the Wilcoxon signed-rank test for paired analysis) in the dosage (median 75mcg/day, range 50-100mcg). No patients were under sex hormone replacement therapy at baseline, except for one male patient with hypogonadism who received a single dose of 250mg of testosterone enanthate 2 weeks prior to baseline measurements. This treatment was immediately discontinued after baseline evaluation. None of the patients with hypogonadism initiated new replacement treatment (testosterone, estradiol/progesterone) until follow-up.

**Figure 1:**
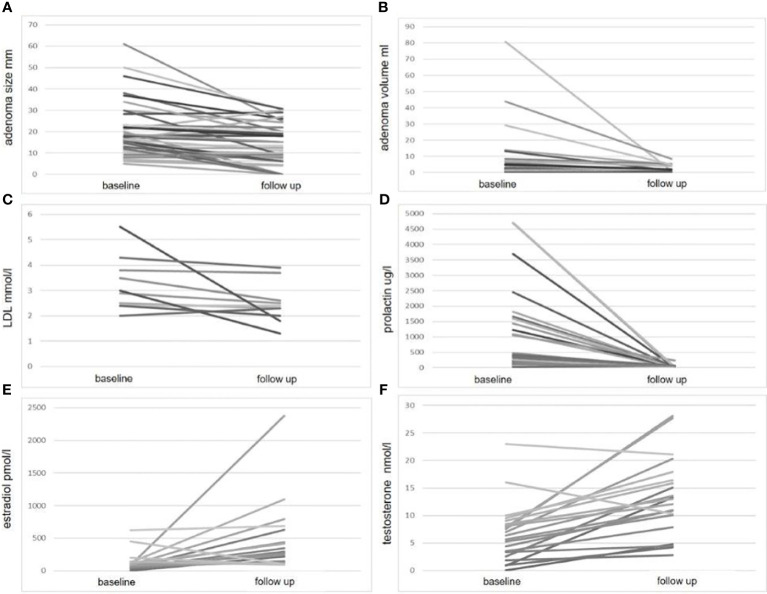
Significant parameter changes at follow-up compared to baseline. This figure illustrates the changes in four parameters: adenoma size **(A)** adenoma volume **(B)** LDL cholesterol **(C)** prolactin level **(D)** estradiol **(E)** and testosterone level **(F)**, measured at baseline and follow-up. For each parameter, individual patient data are plotted on the y-axis, with baseline and follow-up values connected by a line on the x-axis.

**Figure 2:**
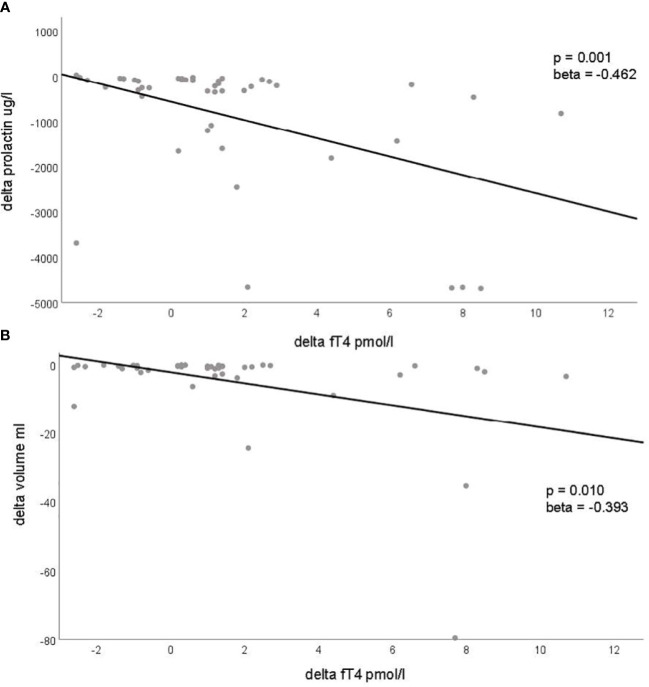
Significant associations with changes (delta) in prolactinoma parameters and changes (delta) of metabolic, inflammatory and endocrine parameters. This figure demonstrates the significant associations identified through regression analysis between changes in prolactin levels [delta prolactin, **(A)**] and changes in tumor volume [delta tumor volume, **(B)**] with changes in fT4 levels (delta fT4). Each dot represents an individual patient, plotting the change in prolactin levels **(A)** and tumor volume **(B)** on the y-axis against the corresponding change in fT4 levels on the x-axis. The regression line is depicted in both panels.

In paired analyses comparing clinical data from baseline to follow-up, we noted a significant reduction in tumor size, volume, and prolactin levels, as expected. Additionally, a decrease in LDL-cholesterol and an increase in estradiol levels in women, testosterone levels in men, as well as an increase in fT4 levels, were observed (**Table 2** and **Figure 1**). No differences were found in the other parameters (**Table 2**). We did not find any correlation between the changes (delta) in LDL and the changes in PRL parameters (**Supplementary Table 4**). However, a negative correlation was observed between the changes in fT4 and the changes in prolactin and adenoma volume (**Supplementary Table 5**). This association was also confirmed as significant in the regression analyses (**Figures 2A, B**; **Supplementary Table 5**).

We performed an additional correlation analysis between the changes (delta) of the endocrine parameters (fT4, cortisol, estradiol, testosterone, IGF-1) and the deltas of the metabolic parameters. The analysis was performed only if data from more than five patients were available for the specific correlation analysis. Besides a significant negative correlation between the change in fT4 and the change in BMI, there was a significant positive correlation between the change in cortisol level and the change in heart rate, as well as a negative correlation with the change in total and LDL cholesterol at follow-up from baseline. No correlation between the changes in testosterone and estradiol levels with the change in metabolic parameters was observed (**Supplementary Table 6**). Moreover, when examining the correlation between baseline PRL parameters and the changes in endocrine, metabolic, and inflammatory parameters, we found a positive correlation only between baseline prolactin levels and the change in fT4 (**Table 3**). This association was also significant in the regression analysis (beta 0.471, p <0.001).

**Table 3:** Correlation analysis between PRL parameters at baseline and the observed difference (delta) of metabolic and inflammatory parameters.

	Adenoma size		Adenoma volume		Prolactin	
	*rs*	*p-value*	*rs*	*p-value*	*rs*	*p-value*
Clinical and metabolic parameters						
Delta BP systolic	0.074	0.648	0.152	0.343	0.115	0.474
Delta BP diastolic	0.113	0.482	0.213	0.180	0.110	0.495
Delta Heart rate	0.155	0.351	0.042	0.801	-0.040	0.811
Delta BMI	-0.166	0.319	-0.142	0.397	-0.269	0.102
Delta HbA1c	-0.253	0.311	-0.081	0.750	-0.332	0.179
Delta Total Cholesterol	-0.174	0.610	0.077	0.821	0.314	0.346
Delta LDL	-0.411	0.238	-0.215	0.551	-0.215	0.551
Delta HDL	-0.515	0.128	-0.297	0.405	-0.588	0.074
Delta Triglycerides	-0.314	0.346	-0.200	0.555	0.127	0.709
Inflammatory parameters						
Delta NLR	0.256	0.189	0.222	0.257	0.041	0.834
Delta PLR	0.264	0.174	0.253	0.194	0.183	0.353
Delta PNI	-0.267	0.230	-0.231	0.302	0.003	0.990
Delta SII	0.210	0.284	0.132	0.503	0.011	0.955
Endocrine parameters						
Delta fT4	0.232	0.125	0.240	0.112	**0.485**	**0.001**
Delta Cortisol	-0.003	0.986	-0.045	0.787	-0.142	0.387
Delta Estradiol	-0.119	0.660	-0.146	0.590	-0.018	0.948
Delta Testosterone	0.214	0.378	0.265	0.273	0.265	0.272
Delta IGF1	-0.370	0.108	-0.303	0.194	-0.094	0.693

Represented are the results of the Spearman correlation between the PRL parameters (adenoma size, adenoma volume, prolactin) and the difference (delta) at follow up from baseline of the clinical/metabolic, inflammatory and endocrine parameters. Results in bold are significant corresponding to a p-value of ≤0.05.

PRL, Prolactinoma; Neutrophile-to-Lymphocyte-Ratio, NLR; Platelet-to-Lymphozyte-Ratio, PLR; Glasgow Prognostic Score, GPS; Systemic Immune Inflammation Index, SII; Prognostic Nutrition Index, PNI; Blood Pressure, BP; Rs, Spearman rho’s.

### 3.2 Predicting outcome

**Figure 3:**
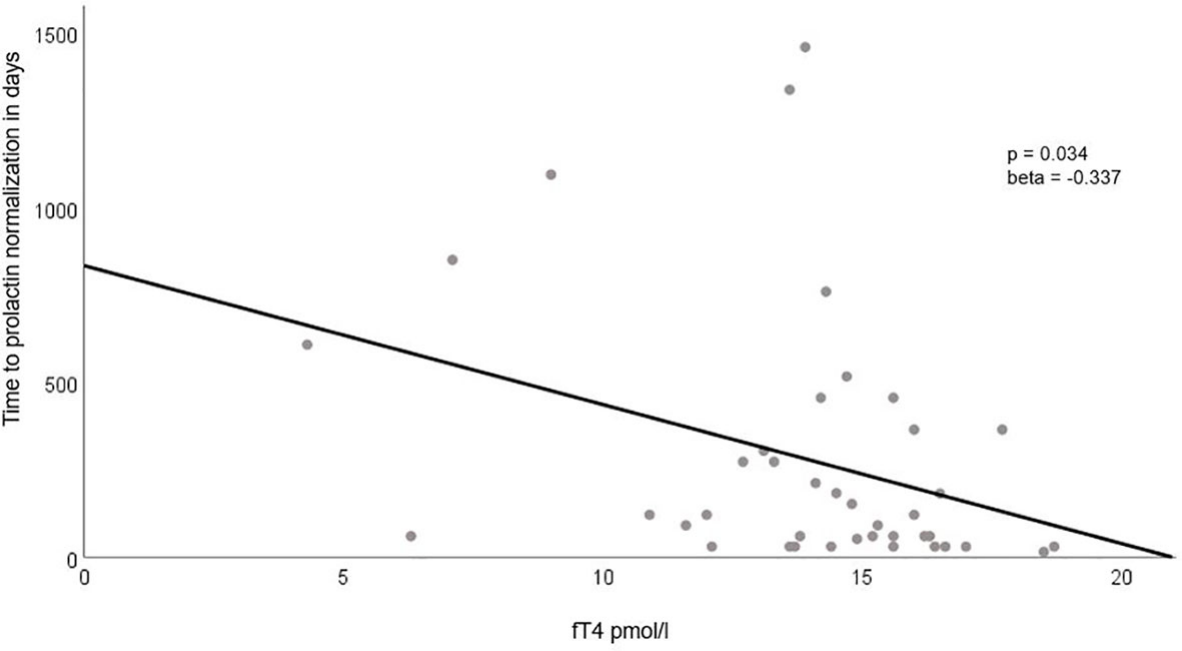
Association Between Baseline fT4 Levels and Time to Prolactin Normalization. This figure highlights the significant negative correlation between baseline fT4 levels (plotted on the x-axis) and the time required for prolactin normalization (presented on the y-axis). The regression line clearly illustrates the inverse relationship between these two variables.

We did not find any significant association between baseline PRL parameters, including clinical, metabolic, inflammatory, or endocrine factors, and the extent of tumor shrinkage at follow-up. This lack of association remained consistent even after adjusting for the time interval between baseline and follow-up MRI scans (**Table 4**). However, we did identify a significant positive association between adenoma size and volume and the time required for prolactin normalization, although this was not the case for baseline prolactin levels. Among the other parameters examined, the only notable finding was a negative association between baseline fT4 levels and the time to achieve prolactin normalization (**Table 4**, **Figure 3**).

**Table 4:** Outcome parameter at follow-up compared to baseline.

	Tumor volume Shrinkage		Adjustment for time interval between MRI		Time to prolactin normalization	
	*beta*	*p-value*	*beta*	*p-value*	*beta*	*p-value*
Parameters at baseline						
PRL parameters						
Adenoma size	0.096	0.541	0.101	0.525	**0.417**	**0.007**
Adenoma volume	0.211	0.174	0.214	0.174	**0.352**	**0.026**
Prolactin (ug/l)	0.267	0.173	0.195	0.226	0.262	0.103
Clinical and metabolic parameters						
BMI (kg/m^2^)	0.110	0.487	0.115	0.472	**0.324**	**0.044**
BP systolic (mmHg)	-0.232	0.155	-0.231	0.163	0.130	0.449
BP diastolic (mmHg)	-0.287	0.077	-0.287	0.080	0.065	0.707
Heart rate (bpm)	-0.056	0.744	-0.056	0.752	-0.243	0.174
HbA1c (%)	0.287	0.220	0.256	0.297	0.152	0.547
Total Cholesterol (mmol/l)	-0.254	0.425	-0.294	0.445	-0.344	0.273
LDL (mmol/l)	-0.231	0.495	-0.254	0.535	-0.342	0.303
HDL (mmol/l)	-0.020	0.953	0.046	0.923	-0.482	0.158
TG (mmol/l)	-0.285	0.369	-0.290	0.387	-0.198	0.538
Inflammatory parameters						
GPS	0.277	0.439	0.277	0.465	0.269	0.399
NLR	0.039	0.838	0.114	0.622	-0.180	0.351
PLR	-0.007	0.970	0.028	0.895	-0.069	0.721
PNI	-0.017	0.936	-0.080	0.718	0.091	0.671
SII	0.022	0.907	0.093	0.692	-0.148	0.443
Endocrine parameters						
fT4 (pmol/l)	-0.075	0.634	-0.064	0.693	**-0.337**	**0.034**
Cortisol (nmol/l)	0.002	0.992	0.025	0.889	-0.169	0.324
Estradiol (pmol/l)	-0.205	0.414	-0.195	0.451	-0.338	0.157
Testosterone (nmol/l)	0.024	0.917	0.026	0.912	-0.383	0.129
IGF1 (ug/l)	-0.049	0.774	-0.063	0.717	-0.170	0.330

Results of the regression analysis between the treatment outcome markers (time to prolactin normalization, tumor shrinkage) as dependent variables with the clinical, metabolic and serum inflammation based scores at baseline as independent variables. ‘Beta’ denotes the standardized regression coefficient. Results highlighted in bold indicate statistical significance, corresponding to a p-value of ≤0.05.

PRL, Prolactinoma; Neutrophile-to-Lymphocyte-Ratio, NLR; Platelet-to-Lymphozyte-Ratio, PLR; Glasgow Prognostic Score, GPS; Systemic Immune Inflammation Index, SII; Prognostic Nutrition Index, PNI; Blood Pressure, BP.

## 4 Discussion

In this study, we present clinical and laboratory data from a retrospective cohort of 59 patients, with additional follow-up data for 46 patients, all from a single tertiary center. We found no correlation or association between inflammatory markers and the clinical/metabolic presentation at baseline or follow-up, as well as with the predefined outcome prediction. However, our PRL cohort displayed several metabolic peculiarities, which have been previously reported in the literature with varying results.

For instance, the prevalence of obesity (31%), overweight/obesity (58.6%), diabetes mellitus (8.5%), and dyslipidemia (20.3%) in our cohort appears to be higher, whilst arterial hypertension (11.9%) lower compared to the general population. According to the Federal Statistical Office in Switzerland, the prevalences in 2022 were 12% for obesity, 43% for overweight/obesity, 5% for diabetes mellitus (excluding prediabetes), 15% for dyslipidemia and 20% for arterial hypertension (45–47). In our analysis, we observed a correlation between increased BMI and tumor size, volume, or prolactin levels, as well as rising HbA1c levels in relation to tumor size and prolactin levels at baseline. Although the regression analysis did not confirm an association between BMI and PRL parameters at baseline, literature supports increased BMI and/or body fat in patients with PRL (48–51), which correlates with baseline prolactin levels (50, 52) and may be independent of hypogonadism (53). Chronic prolactin excess, as seen in patients with PRL, is postulated to directly affect the appetite regulation, leading to increased food intake, contributing to weight gain and even overt obesity in animal models (54). Weight loss post-prolactinoma treatment is documented in several studies but not universally observed [reviewed by (20)]. This effect may be independent of dopamine agonist treatment, as seen in a cohort of surgically treated patients (52). In our cohort, we did not document significant BMI changes, potentially due to the non-standardized timing of follow-up measurements, which may have been too brief to observe such effects.

The impact of prolactin levels on glucose metabolism, indicated by HbA1c levels in our study, was confirmed in our regression analysis. While not statistically significant (p=0.06), we noted a trend towards lower HbA1c levels under treatment. Abnormal glucose homeostasis and higher insulin resistance in patients with PRL, improving after treatment, have been previously described [reviewed by (20, 21)]. The primary effect appears to be due to normalization of prolactin levels, as observed in surgically treated patients (52), but a pharmacological effect is also plausible, as cabergoline treatment improves glucose homeostasis in patients without PRL (55).

Another significant finding in our study is the positive impact of PRL treatment on lipid profiles, similar to the published literature [reviewed by (20)]. We observed no baseline association between PRL parameters and lipids, but a significant reduction in LDL levels post-treatment was noted. This reduction did not correlate with changes in PRL parameters but did with changes in HbA1c. The pathogenesis of this observation is unclear but might relate to changes in BMI and fat distribution as well as improvement of the glucose homeostasis post-treatment, or could stem directly from medical intervention [reviewed by (21)].

However, it remains unclear whether these differences are attributable to prolactin itself or concurrent hypogonadism. It has been hypothesized that the impact of hyperprolactinemia on glucose homeostasis may be direct, through its effects on pancreatic beta cells. This is supported by the discovery of prolactin receptor expression on insulin-secreting cell lines, with chronic hyperprolactinemia also being linked to impaired insulin secretion (21).

High prolactin levels have been shown to directly reduce adiponectin levels in cell and animal model studies. This reduction in adiponectin leads to decreased insulin-mediated inhibition of hepatic gluconeogenesis, resulting in lower glucose uptake and reduced fatty acid oxidation by fat and muscle cells (18, 56).

Within the subgroup of patients with comorbidities, there was a significantly higher proportion of hypogonadism among those with overweight/obesity (94.1% versus 58.3%, p=0.002). The prevalence of hypogonadism in patients with obesity (94.4% versus 72.5%) and dyslipidemia (100% versus 74.5%) was higher, though it did not reach statistical significance (data not shown), likely due to the low total number of cases with the respective comorbidity. However, there might be a direct effect of hyperprolactinemia and its resolution on the lipid profile. Studies in rodents and human adipose tissue cell lines have shown that prolactin directly reduces lipoprotein lipase activity, thereby increasing triglyceride levels (21, 57).

It is crucial to recognize that abnormalities in glucose and lipid homeostasis, as well as fat distribution and BMI, have been described in male patients with hypogonadism (58). Hypogonadism, a major endocrine complication of hyperprolactinemia that usually resolves or at least improves after successful treatment, might play a significant role in the observed metabolic abnormalities and their improvements after successful treatment in patients with PRL. For example, in our cohort, all male patients with hypogonadism had normal gonadal function at follow-up, or at least significantly improved to the extent that no replacement therapy was necessary. Notably, in our cohort, we did not identify any correlation between changes in testosterone levels in males and the metabolic parameters studied. Also in the literature, some studies suggest this beneficial effect after treatment is independent of gonadal function normalization (59, 60).

Other forms of pituitary insufficiency, which were not actively studied in our cohort (for example, we did not perform dynamic testing for growth hormone deficiency or assess for partial corticotrope insufficiency), might also contribute to the metabolic abnormalities in these patients, which might improve or resolve after treatment (61, 62). Interestingly, although we did not observe a significant difference in cortisol levels after treatment, there was a significant correlation between increasing cortisol levels and the reduction of lipid levels (total and LDL-cholesterol) after treatment in our cohort.

However, our study, like others, lacks sufficient patient numbers to fully explore all variables influencing lipid profiles and other metabolic changes in these patients, considering the known potential for multiple pathogenetic mechanisms in PRL patients.

An intriguing observation in our study was the association of fT4 with prolactin parameters at baseline, and a significant increase in fT4 under treatment. The change in fT4 correlated with changes in prolactin and adenoma volume. While no changes in fT4 were documented in the studied literature (20, 21, 48–53, 55, 59, 60), one study reported an increase in fT3 post-treatment with lower baseline fT3 levels compared to controls (51). We found no correlation between fT4 and the metabolic abnormalities observed, beside a negative correlation with the change of BMI after treatment (which was not significant within our cohort as discussed above). Few studies have conducted specific analyses to determine if metabolic changes (BMI, lipid profile) are related to these slight alterations in thyroid hormone production (14, 15, 59). It is known that hypothyroidism leads to increased total and LDL cholesterol, as thyroid hormones regulate the LDL receptor in the liver, reducing LDL clearance in hypothyroidism. Therefore, the improvement in cholesterol levels might also be related to changes in thyroid function.

From the various clinical, endocrine, metabolic, and inflammatory markers tested, we found a positive association between adenoma size/volume and a negative association with fT4 values concerning the time needed for prolactin normalization. We believe that the main reason for the increase in fT4 is related to the decrease in prolactinoma size after treatment. It has been postulated that the mass effect of the prolactinomas causes the partial thyrotrope deficiency observed in these cases, either directly (63) or through an indirect effect on the intrasellar blood flow (64). Due to the small patient cohort, we were not able to definitively assess whether the effect of fT4 was independent of adenoma size and volume, although there was a significant baseline association. Literature suggests male gender is associated with more rapid prolactin normalization (65), but no other parameters have been identified. Similar to one published study [33], we did not find a role for inflammatory markers in the context of outcome prediction.

The major limitations of our study are its retrospective design and the lack of a standardized follow-up protocol, mainly driven by the treating physician’s clinical evaluation and expertise. Additionally, the number of patients included at baseline and with follow-up data is insufficient to identify minor changes or allow for multiple statistical adjustments for factors implicated in the pathogenesis of potential metabolic and inflammatory parameters. Nevertheless, the number of patients in our cohort does not differ significantly from the numbers reported in previously published data (20, 21), which might be the main reason that the differences were not as pronounced in PRL patients compared to those with Cushing disease or acromegaly, where the impact on metabolic and inflammatory traits is more evident (40, 41). However, we believe that our cohort is representative of PRL patients, due to the similarities in our findings related to the metabolic changes also observed in other published studies. We also believe that the lack of findings regarding inflammatory markers might be related to the small sample size in both our study and others.

Another limitation of our study is that we were unable to assess the impact of treatment modality (medical treatment with dopamine agonists versus surgery) on metabolic/inflammatory/endocrine outcomes after prolactin normalization, due to the very low number of surgically treated patients in our registry, which were then excluded from this study. In summary, our study adds further evidence to the metabolic significance of PRL as a disease and negates the role of inflammatory markers in patient stratification and outcome prediction.

## Data availability statement

The original contributions presented in the study are included in the article/**Supplementary Material**. Further inquiries can be directed to the corresponding author.

## Ethics statement

The studies involving humans were approved by Die kantonale Ethikkomission, Zürich. The studies were conducted in accordance with the local legislation and institutional requirements. The participants provided their written informed consent to participate in this study.

## Author contributions

SH: Data curation, Formal analysis, Writing – original draft, Writing – review & editing. LH: Writing – review & editing. SS: Writing – review & editing. RD: Data curation, Formal analysis, Writing – review & editing. LR: Writing – review & editing. CS: Writing – review & editing. FB: Writing – review & editing. ZE: Conceptualization, Data curation, Formal analysis, Investigation, Methodology, Project administration, Resources, Supervision, Validation, Writing – original draft, Writing – review & editing.
