# Supplementary material for: Metabolic and inflammatory parameters in relation to baseline characterization and treatment outcome in patients with prolactinoma: insights from a retrospective cohort study at a single tertiary center
Source: Front Endocrinol (Lausanne). 2024 Apr 5;15:1363939. doi: 10.3389/fendo.2024.1363939 (PMC11026551; doi:10.3389/fendo.2024.1363939)
Supplement: Supplementary file 1 [file DataSheet_1.docx]

Supplementary Material

Metabolic and inflammatory parameters for baseline characterization and treatment outcome of patients with prolactinoma: Insights from a Retrospective Cohort Study at a single tertiary centrum

**Authors:** Susanna Hofbauer, Matthias Ernst, Laura Horka, Samuel Seidenberg, Raffaele Da Mutten, Lucas Regli, Carlo Serra, Felix Beuschlein, Zoran Erlic^1*^

*** Correspondence:**

PD Dr. med. Zoran Erlic

Email: zoran.erlic@usz.ch

# Supplementary Data

**Formula for the calculation of the serum inflammation based scores**

1. The neutrophil-to-lymphocyte ratio (NLR) was calculated by dividing absolute neutrophil by lymphocyte counts.
2. The platelet-to-lymphocyte ratio (PLR) was calculated by dividing absolute platelet by lymphocyte counts.
3. The Glasgow prognostic score (GPS) was based on C-reactive protein and albumin levels (ranging from 0 to 2; gets 1 point to C-reactive protein over 10 mg/dl and 1 to albumin under 35 g/l)
4. The neutrophil-platelet score (NPS) was based on the the leukocyte and the platelet counts: NPS ranging from 0-2 (NPS 0 is defined by neutrophils ≤ 7.5 x10^9^/L and platelets ≤400 x10^9^/L; NPS 1 is defined by neutrophils >7.5 x10^9^/L or platelets >400 x10^9^/L; NPS 2 is defined neutrophils >7.5 x10^9^/L and platelets >400 x10^9^/L)
5. The systemic immune-inflammation-index (SII) was calculcated by multiplication of the absolute platelet count and NLR.
6. The prognostic nutrition index (PNI) was calculated by adding the serum albumin with 5 times the absolute lymphocyte count.

**Formula for the calculation of the prolactinoma volume**

For volume calculation we used the following formula V= 4π/3×1/8×A×B×C=1.05236×A×B×C). B is the maximum length in mm, A the width perpendicular to B and C is at right angles to B and A

|  | **Adenoma size** | | **Adenoma volume** | | **Prolactin** | |  |  |  |  |  |  |  |  |
| --- | --- | --- | --- | --- | --- | --- | --- | --- | --- | --- | --- | --- | --- | --- |
|  | ***rs*** | ***p-value*** | ***rs*** | ***p-value*** | ***rs*** | ***p-value*** |  |  |  |  |  |  |  |  |
| Sex | 0.243 | 0.064 | 0.209 | 0.113 | **0.382** | **0.003** |  |  |  |  |  |  |  |  |
| Age | 0.220 | 0.094 | 0.225 | 0.087 | **0.283** | 0.030 |  |  |  |  |  |  |  |  |

**Supplemental table 1:** Correlations between PRL parameters with sex and age

The results of the correlation analysis between the prolactinoma parameters (adenoma size, adenoma volume and prolactin) with sex and age.

Abbreviations: rs= Spearman rho’s

Results in bold are significant corresponding to a p-value of ≤0.05.

**Supplemental table 2:** Correlations between clinical, metabolic and inflammatory markers with sex and age

|  | **Sex** | | **Age** | |
| --- | --- | --- | --- | --- |
|  | ***rs*** | ***p-value*** | ***rs*** | ***p-value*** |
| ***Clinical and metabolic parameters*** |  |  |  |  |
| BP systolic | 0.160 | 0.248 | **0.432** | **0.007** |
| BP diastolic | 0.124 | 0.372 | **0.300** | **0.028** |
| Heart rate | -0.048 | 0.735 | 0.006 | 0.969 |
| BMI | **0.311** | **0.018** | **0.267** | **0.043** |
| HbA1c | 0.171 | 0.358 | **0.715** | **0.000** |
| Total Cholesterol | 0.163 | 0.470 | **0.476** | **0.025** |
| LDL | 0.148 | 0.521 | **0.499** | **0.021** |
| HDL | -0.150 | 0.527 | -0.239 | 0.311 |
| Triglycerides | 0.111 | 0.622 | **0.432** | **0.045** |
| ***Inflammatory parameters*** |  |  |  |  |
| NLR | 0.194 | 0.218 | 0.280 | 0.072 |
| PLR | -0.102 | 0.520 | 0.125 | 0.430 |
| PNI | -0.071 | 0.683 | **-0.386** | **0.022** |
| SII | 0.042 | 0.792 | 0.215 | 0.172 |
| NPS | 0.126 | 0.431 | -0.127 | 0.429 |
| GPS | -0.104 | 0.681 | 0.093 | 0.715 |
| ***Endocrine parameters*** |  |  |  |  |
| fT4 | **-0.393** | **0.002** | **-0.270** | **0.039** |
| Cortisol | -0.044 | 0.749 | -0.100 | 0.466 |
| Estradiol | -0.022 | 0.920 | 0.140 | 0.514 |
| Testosterone |  |  | -0.338 | 0.068 |
| IGF1 | **-0.318** | **0.022** | -0.303 | 0.194 |

Results of the correlation analysis for the clinical, metabolic, inflammatory and endocrine variables with sex and age.

Abbreviations: rs= Spearman rho’s, BP = Blood pressure

Results in bold are significant corresponding to a p-value of ≤0.05.

**Supplemental table 3:** Correlations between PRL parameters with clinical, metabolic, endocrine and inflammatory baseline markers

|  | **Adenoma size** | | **Adenoma volume** | | **Prolactin** | |
| --- | --- | --- | --- | --- | --- | --- |
|  | ***rs*** | ***p-value*** | ***rs*** | ***p-value*** | ***rs*** | ***p-value*** |
| ***Clinical and metabolic parameters*** |  |  |  |  |  |  |
| BP systolic | 0.167 | 0.229 | 0.084 | 0.544 | 0.058 | 0.678 |
| BP diastolic | 0.208 | 0.130 | 0.103 | 0.459 | 0.062 | 0.657 |
| Heart rate | 0.052 | 0.715 | 0.077 | 0.590 | **0.344** | **0.012** |
| BMI | **0.314** | **0.016** | **0.279** | **0.034** | **0.428** | **0.001** |
| HbA1c | **0.364** | **0.044** | 0.307 | 0.093 | **0.397** | **0.027** |
| Total Cholesterol | 0.343 | 0.126 | 0.201 | 0.371 | 0.157 | 0.486 |
| LDL | 0.345 | 0.118 | 0.177 | 0.443 | 0.191 | 0.406 |
| HDL | 0.021 | 0.928 | 0.049 | 0.838 | -0.040 | 0.867 |
| Triglycerides | 0.376 | 0.085 | 0.318 | 0.149 | 0.237 | 0.289 |
| ***Inflammatory parameters*** |  |  |  |  |  |  |
| NLR | -0.124 | 0.432 | -0.150 | 0.341 | 0.030 | 0.851 |
| PLR | -0.282 | 0.071 | -0.249 | 0.112 | -0.252 | 0.107 |
| PNI | 0.131 | 0.455 | 0.098 | 0.577 | 0.075 | 0.668 |
| SII | -0.131 | 0.410 | -0.141 | 0.374 | 0.011 | 0.945 |
| NPS | 0.187 | 0.241 | 0.200 | 0.209 | 0.161 | 0.315 |
| GPS | 0.206 | 0.411 | 0.206 | 0.412 | 0.383 | 0.117 |
| ***Endocrine parameters*** |  |  |  |  |  |  |
| fT4 | **-0.425** | **0.001** | **-0.401** | **0.002** | **-0.521** | **0.000** |
| Cortisol | -0.219 | 0.108 | -0.167 | 0.224 | -0.089 | 0.519 |
| Estradiol | 0.053 | 0.805 | -0.205 | 0.336 | -0.071 | 0.742 |
| Testosterone | **-0.511** | **0.004** | **-0.570** | **0.001** | **-0.574** | **0.001** |
| IGF1 | -0.169 | 0.232 | -0.175 | 0.216 | -0.175 | 0.215 |

The results of the regression analysis between the significant correlation results with regard to the clinical and metabolic parameters are shown in Table 1B of the manuscript. In the additional regression analysis the association between fT4 with prolactin (beta -0.399. p=0.003), adenoma size (beta -0.411, p=0.001) and adenoma volume (beta -0.288, p=0.024) was confirmed after correction with age and sex.

Abbreviations: rs = Spearman rho’s, BP = Blood pressure

Results in bold are significant corresponding to a p-value of ≤0.05.

**Supplemental table 4:** Correlations analysis between PRL delta parameters and the observed difference (delta) of metabolic and inflammatory parameters

|  | **Delta adenoma size** | | **Delta adenoma volume** | | **Delta prolactin** | |
| --- | --- | --- | --- | --- | --- | --- |
|  |  | |  | |  | |
|  | ***rs*** | ***p-value*** | ***rs*** | ***p-value*** | ***rs*** | ***p-value*** |
| ***Clinical and metabolic delta parameters*** |  |  |  |  |  |  |
| Delta BP systolic | 0.113 | 0.493 | -0.151 | 0.359 | -0.117 | 0.467 |
| Delta BP diastolic | 0.009 | 0.959 | -0.230 | 0.160 | -0.115 | 0.473 |
| Delta Heart rate | -0.313 | 0.063 | -0.196 | 0.253 | 0.028 | 0.869 |
| Delta BMI | 0.259 | 0.133 | 0.259 | 0.134 | 0.285 | 0.082 |
| Delta HbA1c | 0.343 | 0.177 | 0.311 | 0.224 | 0.329 | 0.182 |
| Delta Total Cholesterol | 0.267 | 0.455 | 0.030 | 0.934 | -0.282 | 0.400 |
| Delta LDL | 0.085 | 0.828 | 0.186 | 0.631 | 0.215 | 0.551 |
| Delta HDL | 0.200 | 0.606 | 0.267 | 0.488 | 0.503 | 0.138 |
| Delta Triglycerides | 0.212 | 0.556 | 0.212 | 0.556 | -0.091 | 0.790 |
| ***Inflammatory delta parameters*** |  |  |  |  |  |  |
| Delta NLR | -0.232 | 0.265 | -0.267 | 0.196 | -0.037 | 0.851 |
| Delta PLR | -0.260 | 0.210 | -0.273 | 0.186 | -0.148 | 0.451 |
| Delta PNI | 0.096 | 0.697 | 0.162 | 0.506 | -0.008 | 0.970 |
| Delta SII | -0.288 | 0.162 | -0.240 | 0.249 | -0.008 | 0.967 |

The results of the correlation analysis between the difference (delta) of the prolactinoma parameters (adenoma size, adenoma volume and prolactin) and the delta of the clinical, metabolic and inflammatory parameters.

Abbreviations: rs=Spearman rho’s; BP = Blood pressure

**Supplemental table 5:** Correlations analysis between PRL delta parameters and the observed difference (delta) of endocrine parameters

The results of the correlation analysis between the difference (delta) of the prolactinoma parameters and the delta of the endocrine parameters.

In the additional regression analysis the association between delta fT4 and delta adenoma volume (beta -0.393, p=0.010) and delta prolactine (beta -0.462, p=0.001) was confirmed.

Abbreviations: rs=Spearman rho’s

Results in bold are significant corresponding to a p-value of ≤0.05.

| **Endocrine delta parameters** | **Delta adenoma size** | | **Delta adenoma volume** | | **Delta prolactin** | |
| --- | --- | --- | --- | --- | --- | --- |
|  | ***rs*** | ***p-value*** | ***rs*** | ***p-value*** | ***rs*** | ***p-value*** |
| Delta Prolactin | 0.197 | 0.206 | **0.627** | **0.000** | 1.000 | 0.467 |
| Delta fT4 | -0.277 | 0.076 | **-0.342** | **0.027** | **-0.492** | **0.001** |
| Delta Cortisol | -0.135 | 0.427 | -0.065 | 0.701 | 0.145 | 0.379 |
| Delta Estradiol | 0.021 | 0.940 | 0.264 | 0.341 | -0.026 | 0.922 |
| Delta Testosterone | -0.107 | 0.684 | -0.277 | 0.282 | -0.312 | 0.193 |
| Delta IGF1 | 0.309 | 0.185 | 0.374 | 0.104 | 0.069 | 0.772 |

**Supplemental table 6**: Correlations between differences (delta) in endocrine parameters and differences (delta) in metabolic parameters from baseline to follow-up

|  | **Delta BP systolic** | | **Delta BP diastolic** | | **Delta Heart rate** | | **Delta BMI** | | **Delta HbA1c** | | **Delta Total cholesterol** | | **Delta LDL** | | **Delta HDL** | | **Delta Triglycerides** | |
| --- | --- | --- | --- | --- | --- | --- | --- | --- | --- | --- | --- | --- | --- | --- | --- | --- | --- | --- |
|  | ***rs*** | ***p-value*** | ***rs*** | ***p-value*** | ***rs*** | ***p-value*** | ***rs*** | ***p-value*** | ***rs*** | ***p-value*** | ***rs*** | ***p-value*** | ***rs*** | ***p-value*** | ***rs*** | ***p-value*** | ***rs*** | ***p-value*** |
| Delta fT4 | 0.189 | 0.236 | 0.168 | 0.293 | 0.018 | 0.913 | **-0.370** | **0.024** | -0.176 | 0.485 | 0.132 | 0.699 | 0.055 | 0.880 | -0.309 | 0.385 | -0.191 | 0.574 |
|  | n=41 |  | n=41 |  | n=38 |  | n=37 |  | n=18 |  | n=11 |  | n=10 |  | n=10 |  | n=11 |  |
| Delta Cortisol | -0.18 | 0.286 | -0.120 | 0.478 | **0.464** | **0.006** | -0.020 | 0.913 | 0.334 | 0.206 | **-0.669** | **0.049** | **-0.910** | **0.002** | -0.238 | 0.570 | -0.433 | 0.244 |
|  | n=37 |  | n=37 |  | n=34 |  | n=33 |  | n=16 |  | n=9 |  | n=8 |  | n=8 |  | n=9 |  |
| Delta Estradiol | 0.267 | 0.337 | -0.036 | 0.899 | -0.213 | 0.464 | -0.126 | 0.681 | * | * | * | * | * | * | * | * | * | * |
|  | n=15 |  | n=15 |  | n=14 |  | n=13 |  | n=5 |  | n=2 |  | n=2 |  | n=2 |  | n=2 |  |
| Delta Testosterone | 0.109 | 0.666 | -0.285 | 0.252 | -0.188 | 0.470 | -0.424 | 0.090 | -0.091 | 0.802 | -0.216 | 0.608 | -0.222 | 0.632 | 0.143 | 0.760 | -0.381 | 0.352 |
|  | n=18 |  | n=18 |  | n=17 |  | n=17 |  | n=10 |  | n=8 |  | n=7 |  | n=7 |  | n=8 |  |
| Delta IGF1 | 0.274 | 0.243 | 0.138 | 0.562 | -0.294 | 0.237 | -0.235 | 0.380 | 0.232 | 0.492 | 0.543 | 0.266 | 0.334 | 0.518 | -0.657 | 0.156 | 0.429 | 0.397 |
|  | n=20 |  | n=20 |  | n=18 |  | n=16 |  | n=11 |  | n=6 |  | n=6 |  | n=6 |  | n=6 |  |

Abbrevations: rs=Spearman rho’s, BP = Blood pressure

Results in bold are significant corresponding to a p-value of ≤0.05.

*The number of cases was to low to perform a correlation analysis.
